# Performance improvement of miniaturized ZnO nanowire accelerometer fabricated by refresh hydrothermal synthesis

**DOI:** 10.1098/rsos.170557

**Published:** 2017-09-13

**Authors:** Sangho Song, Hyun Chan Kim, Jung Woong Kim, Debora Kim, Jaehwan Kim

**Affiliations:** Creative Research Center for Nanocellulose Future Composites, Department of Mechanical Engineering, Inha University, 100 Inha-Ro, Nam-Gu, Incheon 22212, Korea

**Keywords:** accelerometer, ZnO nanowire, piezoelectricity, miniaturization, refresh hydrothermal synthesis

## Abstract

Miniaturized accelerometers are necessary for evaluating the performance of small devices, such as haptics, robotics and simulators. In this study, we fabricated miniaturized accelerometers using well-aligned ZnO nanowires. The layer of ZnO nanowires is used for active piezoelectric layer of the accelerometer, and copper was chosen as a head mass. Seedless and refresh hydrothermal synthesis methods were conducted to grow ZnO nanowires on the copper substrate and the effect of ZnO nanowire length on the accelerometer performance was investigated. The refresh hydrothermal synthesis exhibits longer ZnO nanowires, 12 µm, than the seedless hydrothermal synthesis, 6 µm. Performance of the fabricated accelerometers was verified by comparing with a commercial accelerometer. The sensitivity of the fabricated accelerometer by the refresh hydrothermal synthesis is shown to be 37.7 pA g^−1^, which is about 30 times larger than the previous result.

## 1. Introduction

Today's technology trend is towards smaller, lighter and faster. The smaller and lighter one is the faster. Most actuators in haptics, robotics and simulators tend to be small and compact to achieve its high agility [1–3]. To measure the performance of these small devices, a miniaturized accelerometer is necessary because the accelerometer should not influence the weight of the devices. There are several types of accelerometers: capacitive, piezo-resistive and piezoelectric types [4–6]. Capacitive accelerometers measure acceleration by sensing the capacitance change of two electrodes. Capacitive accelerometers typically use a silicon micro-machined sensing element. Their performance is superior in the low-frequency range. Although its structure is simple, two electrodes can be shortened and damaged under impact load. Piezo-resistive accelerometers can measure acceleration by sensing resistance change of the piezo-resistive layer of the accelerometer. Piezoelectric accelerometers rely on direct effect of piezoelectric materials, which has simple structure and wide frequency range. For high sensitivity and miniaturization, the choice of piezoelectric accelerometer is appropriate to evaluate the performance of small actuators [7]. The active piezoelectric layer is beneficial to convert the mechanical signal into electric signal in piezoelectric accelerometers [8]. To fabricate miniaturized piezoelectric accelerometers, the size of the active piezoelectric layer should be reduced. The piezoelectric material should be able to respond sensitively to its external load when it is miniaturized.

Zinc oxide (ZnO), a compound of metal zinc and oxygen, is an important semiconductor material with wide direct band gap of 3.37 eV and a high exciton binding energy of 60 meV. Because ZnO has piezoelectricity, thermal stability, biocompatibility and transparency, it was applied to various industrial fields [9]. ZnO can grow in nanowire, nanosphere and nanotube shape depending on the growing method. Particularly, ZnO nanowire, which is one-dimensional structure, has excellent physical and chemical properties due to high crystallinity and aspect ratio [10]. Miniaturized cantilever-type accelerometers were made by depositing ZnO sensing layer on the cantilever beam [11–14]. Micro-fabrication process was used to fabricate the cantilever structure including tip mass, and ZnO layer was deposited by physical or chemical deposition methods. As miniaturized cantilever-type accelerometers need packaging, they are not valid for measuring acceleration of small and arrayed actuators.

The well-aligned ZnO nanowire can make a high piezoelectric effect through a high strain, making it suitable for the active piezoelectric layer material of the miniaturized accelerometer. Especially, the longer ZnO nanowire is expected to improve the accelerometer performance, because the piezoelectricity increases with increasing the length of ZnO nanowire [15]. Piezoelectric vertically aligned ZnO nanowires were grown by low-temperature aqueous chemical approach and successfully used as a low-frequency self-powered accelerator detector system [16]. The fabricated accelerometer showed a good sensitivity of 4.6 mV g^−1^ in the acceleration range of 0.67–5.5 m s^−2^. However, this sensitivity was obtained under 1 kg head mass, which is impossible for miniaturized accelerometer for small and arrayed actuators.

**Figure 1.**
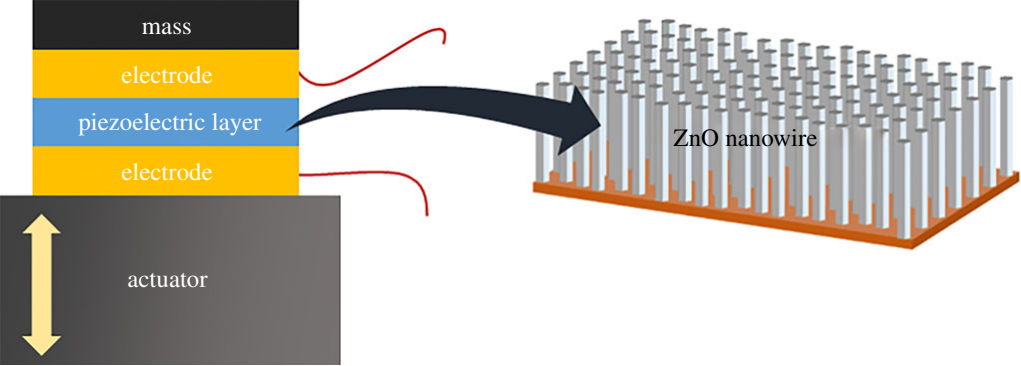
Schematic diagram of the ZnO nanowire accelerometer.

The novelty of this work is to grow long ZnO nanowires uniformly and densely on a copper substrate, so as to fabricate miniaturized accelerometer useful for acceleration sensing of small and arrayed actuators. To further improve the performance of ZnO nanowire accelerometer, we fabricated ZnO nanowire accelerometers by using two hydrothermal synthesis methods for growing well-aligned ZnO nanowires. We expect that the performance of the ZnO nanowire accelerometer can by drastically improved by growing long ZnO nanowires. Performance of the fabricated accelerometer is compared with a commercial accelerometer. Figure 1 shows a schematic diagram of the ZnO nanowire accelerometer.

## 2. Experimental details

### 2.1. Fabrication of ZnO nanowire accelerometer

To fabricate the active piezoelectric layer of accelerometer, a polished copper wafer was prepared as a substrate for growing ZnO nanowire. Copper has a high density of 8.96 g cm^−3^ and an excellent electrical conductivity of 59.6 MS m^−1^ [17]. So, it is useful for the head mass of accelerometer, which applies mechanical load to the ZnO nanowire, and works as the top electrode of the accelerometer.

**Figure 2.**
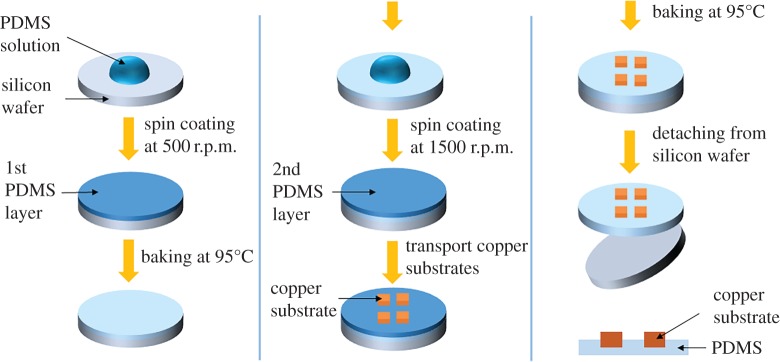
Fabrication process of the PDMS boat.

The copper wafer of 2.5 inch diameter was diced into small square substrates having a volume of 1.5 × 1.5 × 0.5 mm^3^. The copper substrate needs to be in contact with a reaction solution during the hydrothermal synthesis. To float the copper substrate on the solution, a boat was designed by using PDMS (Polydimethylsiloxane, Sylgard® 184, Dow Corning). Figure 2 shows the fabrication process of the PDMS boat. The PDMS and curing agent were mixed in a mass ratio of 10 : 1. The mixed PDMS solution was poured on a 4 inch Si wafer and spin-coated at 500 r.p.m. for 30 s. The first PDMS layer was solidified by baking at 95°C for 5 min. The second PDMS layer was fabricated by spin-coating the PDMS solution onto the first PDMS layer at 1500 r.p.m. The diced copper substrates were transported on the second PDMS layer with the polished side up. The PDMS boat was finished by baking it under the same conditions as the first PDMS layer.

**Figure 3.**
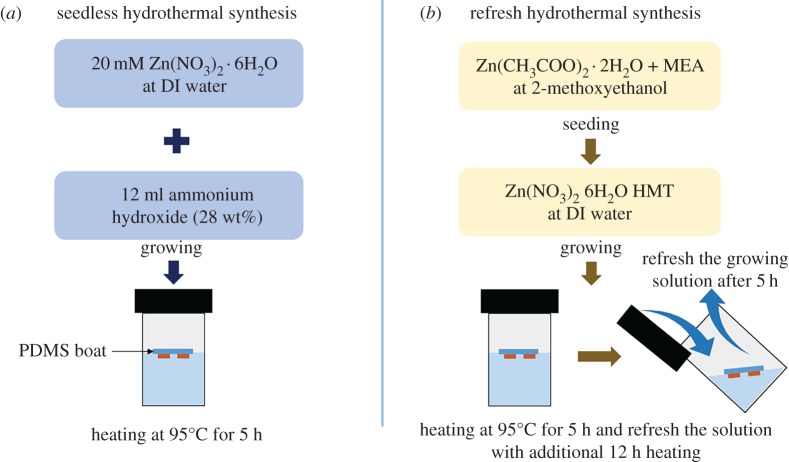
Diagram of the (*a*) seedless and (*b*) refresh hydrothermal synthesis methods.

Two kinds of hydrothermal synthesis were carried out to confirm the performance improvement of the ZnO nanowire accelerometer. Figure 3 shows two hydrothermal synthesis methods. The first method is a seedless hydrothermal synthesis [18]. The reaction solution was prepared by stirring 20 ml of zinc nitrate hexahydrate (Zn(NO_3_)_2_ · 6H_2_O, reagent grade 98%, Sigma-Aldrich) and 12 ml of ammonium hydroxide (NH_4_OH, Sigma-Aldrich) in 35 ml of deionized (DI) water. The reaction solution was filled to 35 ml in a 50 ml vial. The PDMS boat was floated above the reaction solution with the copper substrate facing downward. After that, the reaction occurred at 95°C for 5 h in convection oven.

The second method is a refresh hydrothermal synthesis [19]. The refresh hydrothermal synthesis consists of seeding and growing steps. To fabricate seeding solution, 0.1 M of zinc acetate dehydrate (Zn(CH_3_COO)_2_ · 2H_2_O, reagent grade 98%, Sigma-Aldrich) and 0.1 M of monoethanolamine (reagent plus ≥99%, Sigma-Aldrich) were dissolved in 2-methoxyethanol anhydrous (purity 99.8%, Sigma-Aldrich) and stirred at 60°C for 1 h. The seeding solution was aged at room temperature for 24 h. After that, it was spin-coated on the PDMS boat five times at 3000 r.p.m. for 20 s. The ZnO nanowire growing solution was prepared by mixing 10 mM of hexamethylenetetramine ((CH_2_)·N_4_, reagent grade 99%, Sigma-Aldrich) and 10 mM of zinc nitrate hexahydrate in DI water and stirring for 1 h. The PDMS boat was floated so that the surface of the copper substrate was in contact with the growing solution, and the solution was heated at 95°C for 5 h in convection oven. The exhausted solution was refreshed with new one and the growth of ZnO nanowire was finished by further heating for 12 h.

Two types of accelerometers were fabricated using different length of ZnO nanowires grown by two kinds of hydrothermal synthesis. Two electrodes were required to output the electrical signal of the accelerometer. The copper substrate was used as the top electrode, and silver epoxy was thinly coated on ZnO nanowires to form the bottom electrode. A small amount of silver epoxy was dropped on the host structure and the fabricated ZnO nanowire layer was pressed until the silver epoxy layer securely covers the ZnO nanowire layer. No electrical short contact between the top electrode (copper head mass) and the bottom electrode (silver epoxy layer) was confirmed by measuring resistance between them. Electrical wires were connected to the electrodes to complete the accelerometer fabrication.

### 2.2. Performance test

**Figure 4.**
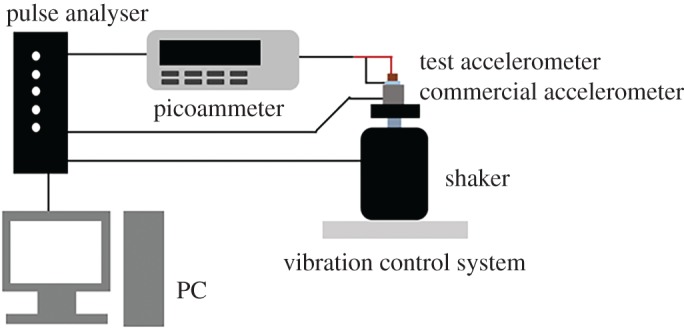
Schematic diagram of the performance test.

To evaluate the performance of the fabricated accelerometers, a comparison test was conducted by using a commercial accelerometer (PCB350c02). The shaker was installed on the vibration control system to apply vibration. The commercial accelerometer was fixed on the shaker and then the fabricated accelerometer was attached on the commercial one. The top and bottom electrode wires of the fabricated accelerometer were connected to picoammeter (KEITHLEY 6485) to collect electrical signal under mechanical vibration. The output current was calculated as RMS (root mean square) value in the frequency domain through pulse analyser (Bruel & Kjaer 35360B-030). The output acceleration of commercial accelerometer was also analysed by pulse analyser. Figure 4 shows schematic diagram of comparison performance test.

## 3. Results and discussion

### 3.1. Fabrication

**Figure 5.**
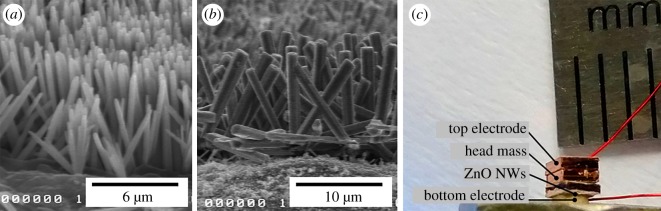
SEM images of the ZnO nanowire on copper substrate by (*a*) seedless and (*b*) refresh hydrothermal synthesis and (*c*) actual image of the fabricated accelerometer.

**Figure 6.**
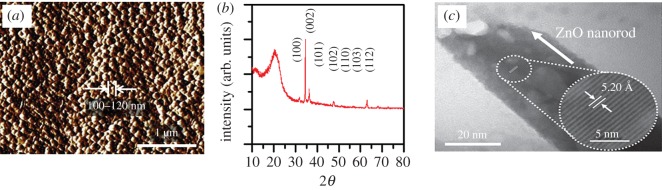
(*a*) AFM image of seeded ZnO particles on cellulose [20], (*b*) XRD pattern of CEZOHN [20] and (*c*) high-resolution TEM image of ZnO nanowire [21].

As results of the seedless hydrothermal synthesis, ZnO nanowire with a length about 6 µm was grown and the refresh hydrothermal synthesis resulted in 12 µm ZnO nanowire, which is twice longer than the seedless hydrothermal synthesis case. Figure 5 shows SEM images of ZnO nanowires grown from two hydrothermal synthesis methods and actual image of the fabricated accelerometer. The averaged diameter of ZnO nanowires is 120 nm with 12 µm length. The ZnO nanowires are shown to be vertically well aligned on the copper substrate. Previously, we have characterized the morphologies and structures of ZnO nanowires grown by the same method by using atomic force microscopy (AFM), transmission electron microscopy (TEM) and X-ray diffraction (XRD) [20,21]. Figure 6*a* shows the previous AFM result of ZnO nanowires. The width of ZnO seeds ranges from 80 to 100 nm with high density and uniformity. The XRD result shown is figure 6*b* indicates that its crystalline is the wurtzite structure, which is beneficial for excellent piezoelectricity. According to the high-resolution TEM result shown in figure 6*c*, the lattice space of ZnO is about 5.2 Å. This lattice space corresponds to the (002) lattice fringe of the wurtzite structure.

### 3.2. Performance test result

**Figure 7.**
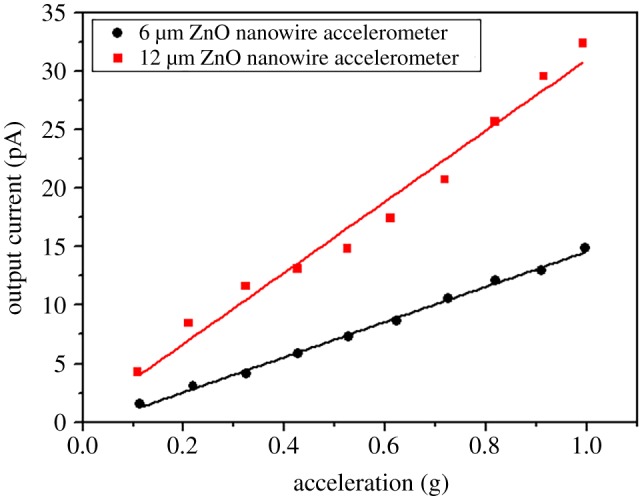
Performance test of the fabricated accelerometers at 100 Hz.

Figure 7 shows the output current of the fabricated accelerometers made with ZnO nanowires by two hydrothermal synthesis methods at 100 Hz. Both accelerometers linearly increased the output current in proportion to the acceleration. The accelerometer with 6 µm ZnO nanowire by the seedless hydrothermal synthesis, increased the output current from 1.5 to 14.9 pA as the acceleration increased from 0.1 to 1.0 g. This indicates that the sensitivity of the fabricated accelerometer is 15.1 pA g^−1^. The accelerometer made with 12 µm ZnO nanowire by the refresh hydrothermal synthesis, increased the output current from 4.3 to 32.4 pA, which indicates that the sensitivity is 37.7 pA g^−1^. This sensitivity is 30 times higher than the previous result (1.1 pA g^−1^) [8]. The accelerometer made with 12 µm ZnO nanowire shows more than twice higher sensitivity than the accelerometer made with 6 µm ZnO nanowire because of the longer length of the ZnO nanowires [8]. Piezoelectric charge of ZnO nanowire is proportionally induced to the length of the nanowire. Thus, as the length of ZnO nanowire increases, the sensitivity of the ZnO nanowire accelerometer increases.

**Figure 8.**
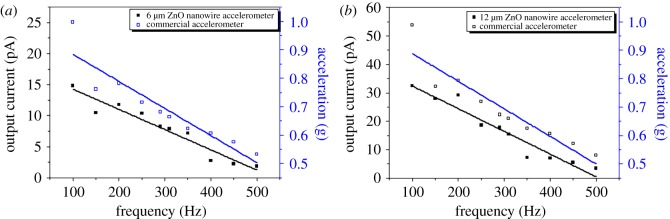
Performance comparison of the commercial accelerometer and fabricated accelerometers with (*a*) 6 µm and (*b*) 12 µm ZnO nanowires.

Figure 8 shows the performance comparison between the commercial accelerometer and the output current of the fabricated accelerometers in frequency domain when a constant voltage was input to the vibration shaker. As the frequency increased from 100 to 500 Hz, the acceleration of commercial accelerometer decreased from 1 to 0.53 g. This phenomenon is natural because under constant input energy of the vibration shaker, the acceleration should be decreased as the frequency increases. The output current of the fabricated accelerometer with 6 µm ZnO nanowire decreased from 14.9 to 1.9 pA and the accelerometer with a 12 µm ZnO nanowire decreased from 32.4 to 3.4 pA. Linear fitting curves shown in figure 8 represent that the slopes of the fabricated accelerometers exhibit the same trend with those of the commercial accelerometer. It is confirmed that the acceleration of the commercial accelerometer and the output current of the fabricated accelerometers have similar behaviour.

**Figure 9.**
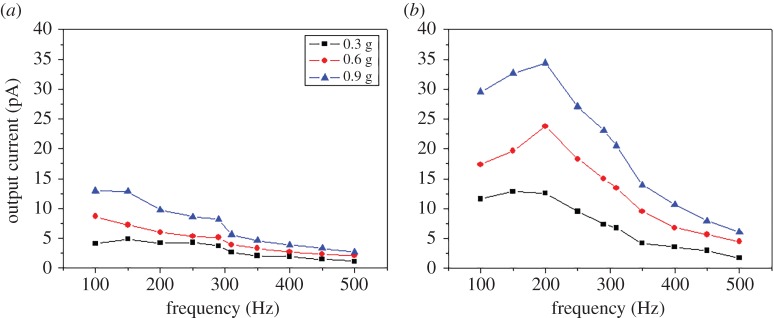
Output current of the fabricated accelerometers with (*a*) 6 µm and (*b*) 12 µm ZnO nanowires.

**Figure 10.**
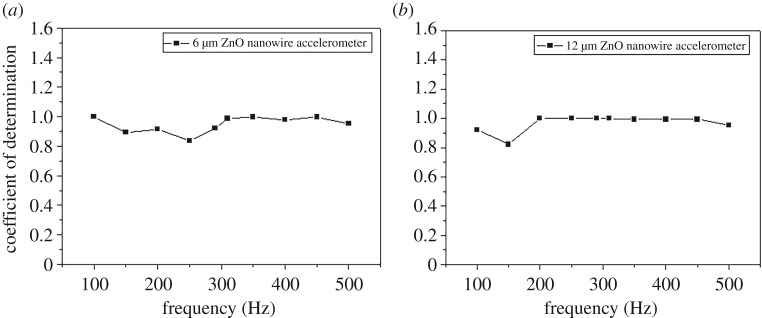
The coefficient of determination for the fabricated accelerometers with (*a*) 6 µm and (*b*) 12 µm ZnO nanowires.

Figure 9 shows the output current of two fabricated accelerometers at constant accelerations of 0.3, 0.6 and 0.9 g. In both accelerometers, the output current value increased proportionally with the acceleration increase for each frequency. In addition, the accelerometer made with 12 µm ZnO nanowire showed three times larger output current value than 6 µm one compared with the same acceleration levels in each frequency. Note that the output current values decrease with increasing frequency, which is natural for dynamics of vibration shaker. Figure 10 shows the coefficient of determination *R*^2^ between the acceleration and the output current for each frequency. The fabricated accelerometer with 6 µm ZnO nanowire shows the coefficient of determination between 0.836 and 0.999 in the operating frequency range and the 12 µm one shows the coefficient between 0.822 and 0.999. These results indicate that the fabricated accelerometers are robust in frequency change.

## 4. Conclusion

Miniaturized piezoelectric accelerometers using ZnO nanowire were fabricated by hydrothermal synthesis, and performance was evaluated with a commercial accelerometer. The seedless and the refresh hydrothermal synthesis methods made 6 µm and 12 µm ZnO nanowires, respectively. The fabricated accelerometers linearly produced electrical signal output from externally applied vibration. The fabricated accelerometer by refresh hydrothermal synthesis showed higher sensitivity than the seedless, with a wider electrical signal output range. The sensitivity of the accelerometer made with 12 µm ZnO nanowire by the refresh hydrothermal synthesis was 37.7 pA g^−1^. This sensitivity is 30 times higher than the previous result (1.1 pA g^−1^). The ZnO nanowire accelerometer made by refresh hydrothermal synthesis is useful for fabricating miniaturized accelerometer.

## Data accessibility

The datasets supporting this article are available at Dryad: (http://dx.doi.org/10.5061/dryad.55636) [22].
